# Worsened multitasking performance with simulated hearing protection correlates with individuals’ choice not to “wear” it

**DOI:** 10.3389/fpsyg.2026.1657161

**Published:** 2026-06-09

**Authors:** Matthew G. Wisniewski, Patricia McClain, Chelsea N. Joyner

**Affiliations:** 1 Department of Psychological Sciences, Kansas State University, Manhattan, KS, United States; 2 Children’s Mercy Hospital, Kansas City, MO, United States; 3 Department of Psychology, Emporia State University, Emporia, KS, United States

**Keywords:** hearing protection, listening effort, occupational hearing loss, safety science, speech-in-noise

## Abstract

**Introduction:**

Workers’ reasons for not wearing hearing protection devices (HPDs) largely come from performance issues that occur while wearing. Research has shown that such complaints are justified in regard to auditory situational awareness (e.g., speech comprehension), but other aspects of work performance (e.g., multitasking ability) are rarely characterized. Further, the relationship between HPD-related performance worsening and the choice to wear an HPD has not been objectively characterized. We address both gaps in the literature here.

**Methods:**

Participants were presented with moving objects on a computer screen and tasked to track a “target” object by hovering a cursor over it. Target objects where identified by auditory commands sourced from the Coordinate Response Measure (CRM) corpus sentences of the form: “*Ready < call sign > go to < color > < number > now*”. The color/number pair indicated the target (e.g., blue four = blue square on screen). CRM sentences were masked by background environmental noise, with sounds either left unaltered or digitally filtered to simulate HPD wearing. After completing 8 blocks alternating between no HPD and HPD conditions, participants were given the option to choose whether or not to “wear” the simulated HPD in an additional block that could earn them bonus pay for their participation. They were then queried as to how much pay they would need to make the opposite choice (e.g., to choose to wear the HPD if they initially chose not to wear it).

**Results:**

The results show that wearing an HPD can have a significant negative impact on aspects of job performance that include auditory situational awareness (reflected in detecting and tracking the correct target objects) and sensorimotor tracking ability (reflected in how tightly they tracked moving targets). Also, clear preference to listen in the no HPD condition was seen when payment depended on performance.

**Discussion:**

We believe that performance problems associated with HPD wearing (both auditory and non-auditory) should be addressed if real workplace change in HPD use rates is to occur.

## Introduction

The wearing of hearing protection devices (HPDs) can prevent occupational hearing loss (HL) in high-noise work environments (Gallagher et al., 2014). However, compliance in wearing HPDs is much lower than desired (Arezes and Miguel, 2005; Green et al., 2021). This contributes to an increasing prevalence of occupational HL as a worldwide health problem (Hasson et al., 2011; Mathers and Loncar, 2006). Factors that can keep workers from wearing HPDs include: safety culture of the workplace (Cavallari et al., 2019), access (McCullagh et al., 2002), HPD comfort (Davis, 2008), knowledge of occupational HL as a consequence of noise exposure (Arezes and Miguel, 2005), knowledge of how to properly wear an HPD (Hager, 2011), and work performance issues that arise while wearing (Casali et al., 2009; Gallagher et al., 2014; Morata et al., 2001; Nelson et al., 2020). Some of these factors are actively being addressed in hearing loss prevention programs (HLPPs; for review see Brennan-Jones et al., 2020; Rabinowitz et al., 2018). For instance, the Occupational Safety and Health Administration (OSHA) has published guidelines for HPD fit testing, employer requirements to provide HPDs, repeated audiogram evaluation for workers, worker training on noise exposure issues, and monitoring of noise levels (Occupational Health and Safety Administration, 2002). Even so, systematic reviews examining the impacts of HLPPs that use these recommendations has revealed “low quality” evidence of benefits (Brennan-Jones et al., 2020; Tikka et al., 2017). HPD non-use prevalence has remained high (∼53% in noise-exposed workers) even after the OSHA guidelines were put in place (Green et al., 2021).

There has been a recent interest in better characterizing performance detriments while wearing HPDs. This is because worsened work performance (broadly construed) is often stated by workers to be a main reason they choose not to protect themselves (Casali et al., 2009; Couth et al., 2022; Morata et al., 2001; Nandi and Dhatrak, 2008; Olson et al., 2016; Reddy et al., 2014). The vast majority of research has examined the natural trade-off between HPD attenuation of sound and the ability to extract information from auditory environments. Unsurprisingly, wearing of an HPD generally degrades auditory situational awareness. For example, speech perception is worse while listening with an HPD than listening with an open ear (Gallagher et al., 2014; Smalt et al., 2020; Wisniewski and Zakrzewski, 2020). Detection and localization of auditory alarms at work can also be worsened by HPD wearing (Arz et al., 2018; Laroche et al., 2022). Non-auditory aspects of work performance have been less often characterized, but could also be a strong barrier to increasing HPD use rates. There is current experimental evidence for the wearing of an HPD causing worsened sensorimotor tracking ability (Wisniewski and Chuwonganant, 2025), slower reaction times (Smalt et al., 2020), and worsened memory performance (Wisniewski and Zakrzewski, 2020). These dual-task performance problems can be seen even when individuals show accurate speech comprehension, leading many researchers to use performance cost as a measure of listening effort (for review, see Gagné et al., 2017). All these aspects could hinder performance-based employment opportunities and career progression for a worker.

Our first goal of this study was to characterize how HPD wearing impacts multiple aspects of work performance in a task where auditory situational awareness, sensorimotor tracking, and visual change detection can be evaluated in parallel (Wisniewski and Chuwonganant, 2025). In our previous experiments that developed this task, listeners heard auditory commands from the Coordinate Response Measure (CRM) corpus (Bolia et al., 2000) telling them the identity of a target object to track on a screen with a computer mouse. We found that both simulated and real HPD listening negatively impacted speech comprehension overall (shown by the number of frames spent tracking the correct target object), but only negatively impacted sensorimotor tracking accuracy during critical listening periods of the task (i.e., during CRM sentence presentation). Here, we expand on this paradigm by also examining the ability to adapt to changes in target object movement direction. We also have subjects confirm finding the target object by clicking on it for a more direct measure of speech comprehension accuracy. The result is the most detailed objective characterization of HPD impacts on non-auditory aspects of work performance to date.

Our second goal was to perform an initial examination of the relationship between performance and choice-to-wear an HPD. Though workers often report that performance detriments are a reason for not wearing an HPD, controlled investigations of objectively measured choice are non-existent. Here, we told listeners that they could earn cash payment for one more block of participation after all actual experimental blocks were completed. We gave them a choice to listen in the HPD condition (referred to as “quiet” to the listener) or the no HPD condition (referred to as “loud” to the listener). After making this choice we also asked how much bonus pay they would need in order to choose the opposite. We predicted that listeners would be more likely to choose the no HPD condition, and that the value of this choice would be related to individual differences in their task performance.

## Materials and methods

### Participants

Participants were 68 individuals from the Kansas State University community that participated for $15 or for course credit. Participants were told that they could also win a small monetary award. One participant was removed from analysis due to a computer error that led to poor audio-visual synchronization. Another was removed randomly to maintain block order counterbalancing, leaving a sample size of *N* = 66. All participants self-reported normal hearing. There was no audiological testing. Average age was 20.17 years (SD = 4.62). Forty-five participants were female.^1^ Sample size was determined based on our previous experiments in which the observed difference between HPD and no HPD conditions for the number of tracked video frames would yield ∼80% power at a sample size of 22. Further assuming that a meaningful correlation between choice and performance would be *r* = 0.35, the obtained number of participants achieves over 80% power for both potential effects.

### Equipment and apparatus

Sounds were presented over RefTone LD-3 speakers (Reftone, Woodland Hills, CA) at ∼15° ± azimuth angle from a listener situated in a WhisperRoom sound attenuating booth (WhisperRoom, Knoxville, TN). The listener sat in a chair ∼2′ from the speakers. Speakers were powered by an ART SLA-2 (ART Pro Audio, Niagara Falls, NY) amplifier and fed by signals from an RME UC Fireface audio interface (RME, Germany). The speakers played identical signals at an average level of ∼70 dBA for the no HPD condition across all blocks. This level was selected to match that of the employed background street recording used for masking (see below). Listeners used a computer mouse to track objects on a 24″ LED computer monitor. All procedures were programmed in MATLAB (Mathworks, Natick, MA) and used Psychophysics Toolbox (Brainard, 1997) combined with custom MATLAB code.

### Stimuli and task

A more detailed description of task development (e.g., recording of background sound, CRM sentence timing, digital HPD filter simulation design, etc.) are given in Wisniewski and Chuwonganant (2025). The stimuli built for that study were used here, and are freely available at https://osf.io/dg8za/. This dual task type of paradigm was designed to mimic features of many occupational tasks that require a listening and a manual motor control component (e.g., operating machinery with voice instructions while being exposed to loud noise). We will describe the most pertinent details below.

A recording of street noise at a busy intersection in Manhattan, KS was used for continuous masking of CRM sentence presentations. The CRM contains sentences of the form: “*Ready < call sign > go to < color > < number > now*” (Bolia et al., 2000). Possible call signs included Baron, Charlie, Ringo, Laker, Arrow, Tiger, Eagle, and Hopper. All eight talkers in the corpus were used (4 female). Color possibilities were limited to Red, White, and Blue. Number possibilities were limited to Three, Four, and Five. CRM sentences were randomly generated with a 10% probability of a sentence containing a critical “Baron” call sign. Eight five-minute audio excerpts were constructed by combining these CRM corpus sentences. Individual sentences were separated in their onset by 1 - 4 s, and the level of each sentence was roved from −6 dB to 0 dB relative to the average level of the background street noise.

To simulate listening with an HPD, Wisniewski and Chuwonganant (2025) designed a digital filter with an attenuation profile that matched that of a Howard Leight Max passive HPD. In general, the filter had a low-pass profile with a flat ∼30 dB attenuation from DC to ∼100 Hz. Many other types of passive hearing protectors have similar low-pass profiles (Gallagher et al., 2014). In the HPD condition for the current study, sounds were digitally filtered with the same filter before presentation to the listener.

**FIGURE 1:**
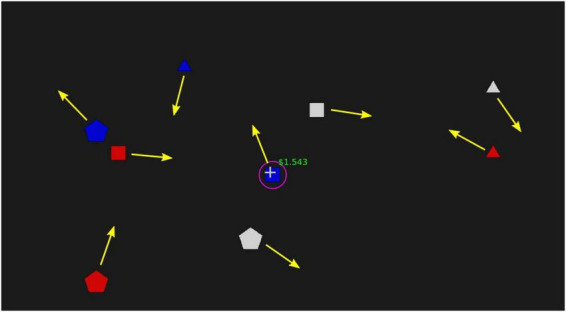
Example video frame. This figure shows an example video frame from the task with corresponding object movement directions (yellow arrows), a Euclidean distance cutoff of 50 pixels from the target object’s center (pink circle), and feedback to the subject in regard to earned dollars. The yellow arrows and pink circle are for display only. They were not shown to subjects.

For the tracking component of the task, videos were created where shapes on screen could be colored red, white, or blue. These shapes could have 3 (triangle), 4 (square), or 5 (pentagon) sides, making a total of nine different objects. Objects moved in randomly determined vectors across screen at a rate of 7 pixels per video frame at 30 frames per second. Shapes would change directions along a new vector at random. Figure 1 shows an example video frame. A cross is shown at the cursor location for the example video frame. The pink circle shows a Euclidean distance of 50 pixels used as a cutoff for gaining hypothetical dollars with accurate tracking (see below). This cutoff was determined in pilot testing to be within the normal range of sensorimotor tracking ability for most subjects. The yellow arrows describing movement direction and the Euclidean distance borders are only for display. They were not seen by subjects.

Recorded street noise, CRM sentence streams, and videos were combined to make eight separate excerpts (for more details, see Wisniewski and Chuwonganant, 2025). Upon hearing a “Baron” call sign sentence, listeners were instructed to find the target object indicated, click on it, then track the object within 50 pixels to gain hypothetical dollars. Dollars were only gained after a click of the correct target object was made. When the target object was tracked within 50 pixels, an earned dollar amount displayed beside the cursor in green and increased at a rate of $0.001 per video frame. When the cursor was outside this window, or the correct target object was not clicked yet, the earned dollar amount was displayed in red and remained unchanged. Upon presentation of each “Baron” call sign sentence, the target object switched immediately after the word “now.” At the end of each block, listeners were shown how many hypothetical dollars they earned.

Before starting the first block, listeners were given training in a simplified version of the task. This simplified version used the no HPD condition to make sure that participants understood the task’s demands. The training consisted of three short blocks that progressed from slowly moving objects (1 pixel per frame) with only “Baron” sentences, to a block with distracting non-“Baron” sentences, to a final block with all sentence types and objects that moved at the pace of the actual experiment. The experimenter monitored participant performance in order to make sure they understood the task. All participants who moved onto the actual experiment were able to demonstrate that they could track a majority of frames in the final block of this simplified task.

Half of the blocks in the actual experiment presented sounds with no filtering (no HPD condition), while the other half presented sounds that were filtered (HPD condition). Block type alternated across the eight block session. Half of the listeners performed the HPD condition on the odd numbered blocks. Individual excerpts were randomly assigned to blocks for each subject.

After the completion of eight blocks, listeners were instructed that there was one more block in which their performance would determine the dollar amount they would be given for compensation. A GUI was then presented on screen in which the listener could choose which condition they would like to listen in: loud (no HPD) or quiet (HPD). After their selection, another GUI was presented where participants were asked: “How much bonus pay would you need to select the < opposite > condition?.” Response options ranged from $0 to $10 in $1 increments. No additional block was run in actuality. Subjects were informed of this deception in debriefing.

### Measures and statistics

**FIGURE 2:**
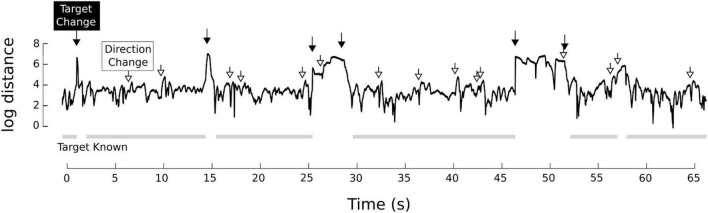
Example of how performance measures were extracted from the temporal dynamics of tracking. The solid black trace shows the log distance between the target object and the cursor. The gray line underneath marks frames of the data that were classified as the target object being known. Switches in the target object are represented by solid arrows. Switches in target object direction are represented by open arrows.

We intended to look at task performance measures that characterize speech comprehension and sensorimotor tracking independently of each other. Because dollars earned in the task reflects both of these aspects, we analyzed instead the number of video frames in which the target object was known, and the Euclidean distance between the target object’s center and the cursor. Figure 2 depicts how these measures were extracted based on a representative example of data from a single subject.

For the number of frames measure, a frame was defined as the target object being known if: (1) the target object was clicked, and (2) the cursor was within 150 pixels of the target object. The 150 pixel cutoff was adopted from our previous paper (Wisniewski and Chuwonganant, 2025). The distance measure was included in this determination so as not to corrupt the measure by potential false alarm switches to tracking other non-target objects. The gray shading underneath the solid black trace in Figure 2 shows the result of this classification for the representative sample. For the distance measure we used the log transform of Euclidean distance between the center of the target object and the cursor for only those video frames classified as times when the target object was known. The transform was used because of a positively skewed distribution for the distance variable. With this method, we were able to separate the influences on tracking that were caused by poor speech perception from influences related to other potential factors (e.g., increased cognitive load). For instance, see the first and third target changes. The first shows a relatively quick transition back to tracking, while the third shows that the participant fails to find the indicated target object until the next one occurs. For the third target change, the large tracking error is a result of poor speech comprehension of the previous CRM sentence, not poor tracking ability.

The number of frames determined to be classified as tracking for each block and the median of the long distance measure across frames were entered into separate linear mixed effects model analyses. These models were fit in MATLAB’s statistics toolbox using maximum likelihood estimation. Distributions of residuals were visually inspected for violations in normality. None were observed. Block (centered and continuous), HPD condition (categorical), and their interaction were included as fixed effects. The random effects structure included subject intercepts and slopes for the fixed effects, and an intercept for the excerpt presented for a block. The latter served to account for the possibility that different excerpts could potentially have different degrees of difficulty. The significance of each fixed effect was assessed with a likelihood ratio test comparing the full model with that of a reduced model having the effect of interest removed. Beta coefficients from the full model were inspected to interpret any significant effects.

To characterize how sensorimotor tracking performance varied as a function of task events, we created epochs of tracking error from −5 s to +4 s surrounding target object switches (solid arrows in Figure 2) and changes in target object direction (open arrows in Figure 2). These epochs were then trimmed to those in which a subject had all time frames classified as having a known target object from −5 s to −4 s and from +2 to +4 s. This was to ensure that we were analyzing only those times in which the target object was known before a change and soon after a change. The median of these epochs was then used to make up an individual’s event-related tracking vectors. A non-parametric permutation approach was taken to assess significance between the HPD and no HPD conditions. We constructed a null hypothesis distribution by randomly reassigning HPD condition labels to individual epochs and recreating event-related tracking vectors. This was performed for 10k iterations. A *p*-value for each time point was then determined based on the proportion of null hypothesis iterations in which the average difference between the HPD and no HPD conditions was more extreme than the observed data. These *p*-values were interpreted with the false-discovery rate procedure to limit the family-wise error rate to 0.05 (Benjamini and Hochberg, 1995).

To analyze the choice data, we conducted a χ^2^-test to determine whether any unbalanced choice for either the HPD or no HPD condition was significant. To assess the value of this choice as it related to performance, we used Pearson correlations to determine if there was a relationship between dollars earned in the task and the bonus pay that subjects indicated they would need to select the opposite condition. This was done separately for those choosing an HPD and those choosing no HPD.

## Results

### Wearing an HPD worsens speech comprehension

**FIGURE 3:**
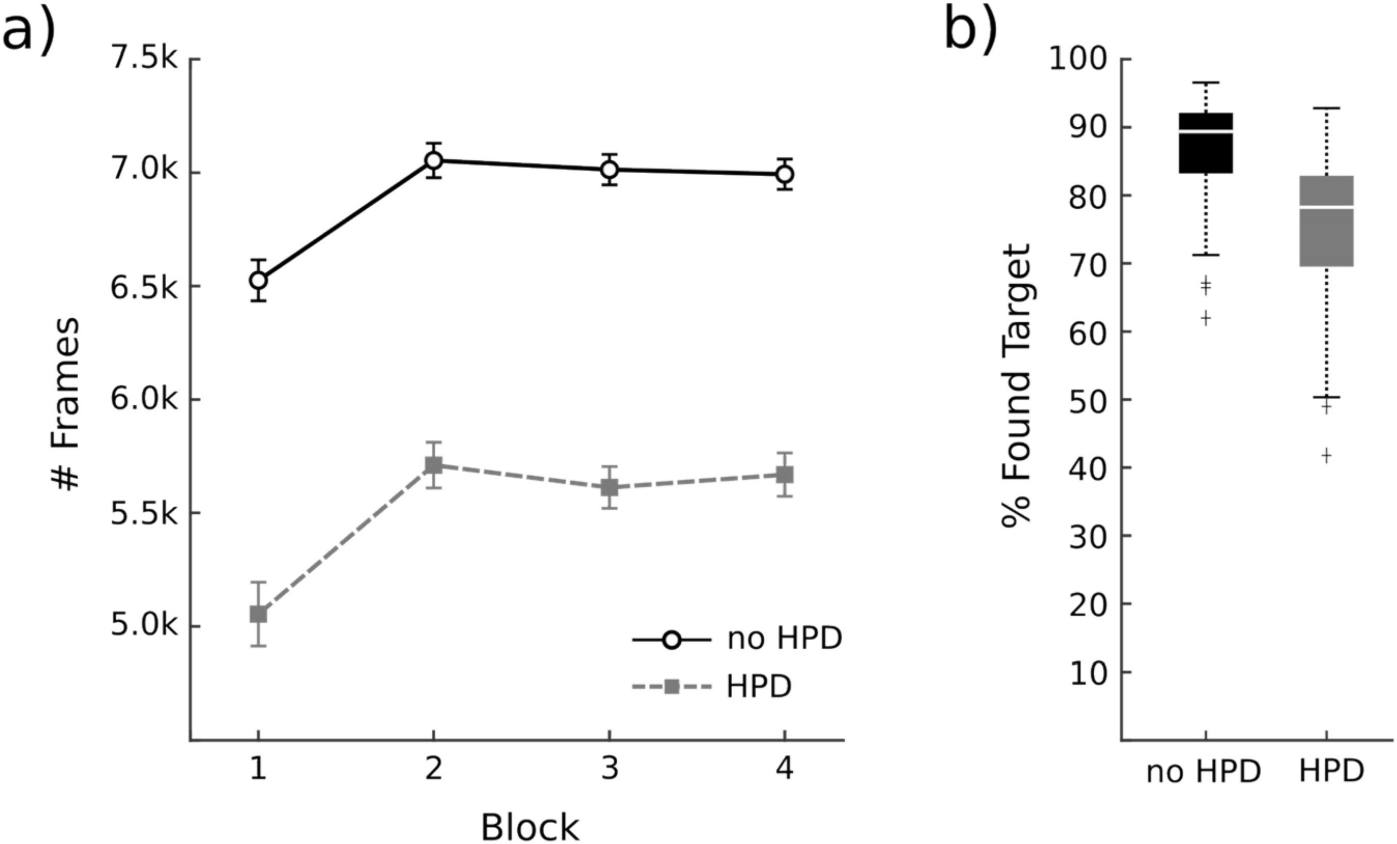
Video frames spent tracking the target object and % of target objects found. **(a)** Average number of video frames in which the target object was known to subjects in the no HPD and HPD conditions for each block. Error bars show within-subject standard errors of the mean (Cousineau, 2005). **(b)** Boxplots showing median, inter-quartile range, range, and outliers (+) for the % of target object changes found within 2 s.

Figure 3a shows the average number of video frames per block in which the target object was known (i.e., a click was registered and the cursor was within 150 pixels of the target object). There is a clear impact of simulated HPD wearing, with the no HPD condition showing more frames known, χ^2^(1) = 125.75, *p* < 0.001, *β_*HPD*_* = −1411.60, 95% CI = [−1558.30, −1265.00]. We expect that much of this effect has to do with not comprehending the CRM commands prior to a target object change for the HPD condition. To more formally assess this, we compared the proportion of target object changes in which a correct click was registered within 2 s following the change for HPD and no HPD conditions, presuming that new target object identification in this time window reflects correct comprehension of the command. A nonparametric Wilcoxon sign-rank test was used. Indeed, the no HPD condition had a higher proportion of found target objects within this time period (Figure 3b), *z* = 7.04, *p* < 0.001.

Further regarding the number of frames tracked, there was a significant effect of block, χ^2^(1) = 10.38, *p* = 0.001, *β_*Block*_* = 133.75, 95% CI = [54.63, 212.87]. This is a learning effect, showing that listeners get better at tracking the correct target object with repeated practice. The HPD condition x Block interaction was nonsignificant, *p* > 0.50.

### Wearing an HPD worsens sensorimotor tracking

**FIGURE 4:**
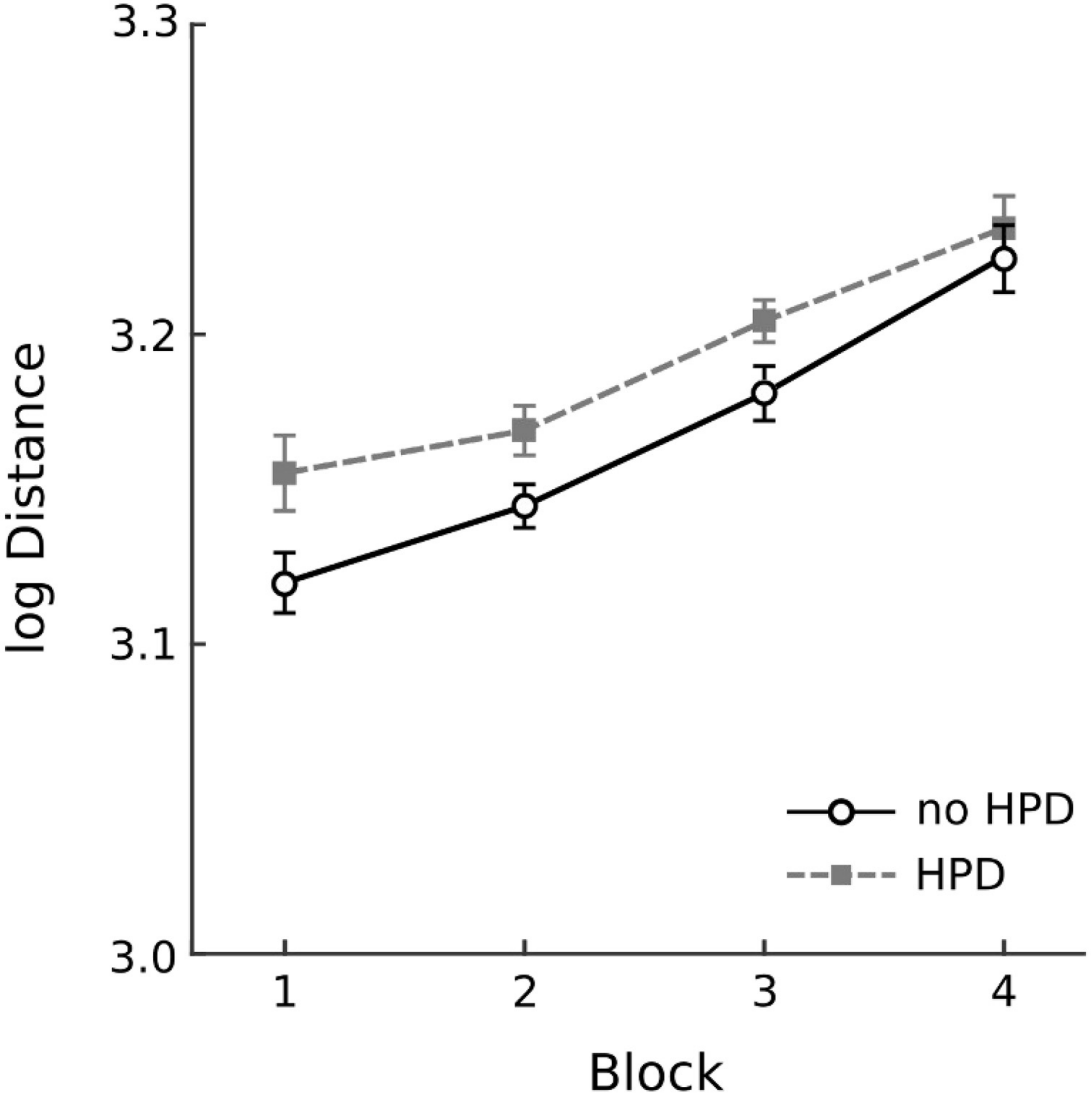
Tracking error for known target objects. Average log distance between the cursor and the center of the target object on frames classified as the target object being known. Error bars show within-subject standard errors of the mean (Cousineau, 2005).

Figure 4 shows the subject average of log median distance between the cursor and the center of the target object for only those data points classified as the target object being known. There was a significant effect of HPD condition, χ^2^(1) = 12.55, *p* < 0.001. This was such that sensorimotor tracking was less accurate when a subject was listening in the HPD condition, *β_*HPD*_* = 0.007, 95% CI = [0.004, 0.011]. There was also an effect of Block, χ^2^(1) = 27.37, *p* < 0.001, showing worsened tracking performance as a session reached its end, *β_*Block*_* = 0.011, 95% CI = [0.007, 0.015]. This could be related to fatigue during the ∼ 1 h session. The interaction effect did not reach significance, *p* > 0.05.

**FIGURE 5:**
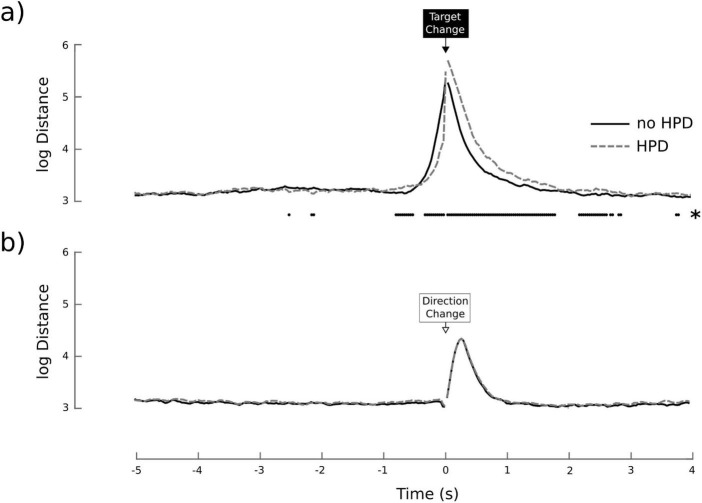
Temporal dynamics of tracking error. **(a)** Average of tracking error epochs time-locked to changes in the target object (i.e., the end of CRM sentences with the “Baron” call sign. **(b)** Average of tracking error epochs time-locked to changes in target object direction. Black points below the traces mark significant differences between the HPD and no HPD conditions (*p < 0.05; FDR corrected).

Figure 5a shows tracking error, time locked to the end of critical “Baron” call sign sentences. At time 0, the target object is switched and dollars are only gained if this next object is clicked and the cursor hovers over it from a distance ≤ 50 pixels (see Methods). Significant differences between the HPD and no HPD conditions (marked below traces of average tracking error) arise strongly just prior to the target object change (during CRM sentence presentation) and sustain during the search the next target object. The direction of this difference is related to time relative to the change. Initially, tracking is worse for the HPD condition (starting at ∼-1 s). This replicates our previous work. Interestingly, this trend shows a reversal just prior to the target object change. This likely reflects a faster search initiation under the no HPD condition. A quicker processing of the speech corresponding to the new target could allow participants to move earlier, which shows up as worse tracking performance because they have moved away from the target object before the word “now” at the end of CRM sentences. In line with this, subjects appear to find the new object faster under the no HPD condition than the HPD condition. This is exemplified by the significant differences between HPD conditions after the change point.

Figure 5b shows tracking error time locked to the onset of changes in target object direction at times when the target object was known. It was expected that there would be a jump in tracking error around changes in direction because it should take time for participants to adjust to new movement directions. This was indeed the case. However, no significant differences between the HPD conditions was found at or near changes in target object direction.

### Performance impacts of HPD wearing relate to “choice-to-wear”

To assess choice-to-wear after listeners experienced both the no HPD and HPD conditions, we first analyzed the choice to listen in the “silent” (HPD) or “loud” (no HPD) condition in one final block that the listener was told could earn them actual dollars in the experiment. It was the case that 84.8% of listeners chose “loud,” corresponding to the unprotected no HPD condition. This value was significantly different from chance, χ^2^(1) = 32.06, *p* < 0.001. This makes sense as the choice does not require from the subject an evaluation of how damaging not wearing an HPD could be. Nevertheless, the data does show objectively that choice to wear an HPD is related to the performance detriments that most HPDs will inevitably produce.

The average dollar amount selected for a bonus reward was around $7 (*M* = 6.68; *SD* = 2.90). The award amount selected was not significantly related to individuals’ dollars earned within blocks of the actual experiment for either those choosing to listen with, *r*(8) = 0.48, *p* > 0.15, or without an HPD, *r*(54) = −0.05, *p* > 0.73. There thus didn’t appear to be any indication that individual performance in the task was related to the value placed on an individuals’ choice.

## Discussion

The current study has demonstrated a number of important features of HPD wearing in regard to performance and individuals’ choice to “wear” an HPD. There are many jobs that depend on simultaneous listening in loud environments, fine motor skill, and visual attention that are mimicked in our paradigm. Instances include machinery operators (e.g., forklift control), warfighters (e.g., targeting with weapons systems), first responders (precise medical skill at an accident site), and musicians (e.g., controlling minutiae of instrument tonality). The data bolsters recent calls to consider that workers may choose not to wear hearing protection because it leads to poor performance at work.

We show that performance detriments can make themselves apparent in a number of ways. Auditory situational awareness is only one. In our study, auditory situational awareness was best characterized by the number of video frames spent tracking the target object while wearing an HPD, and the proportion of times in which the target object was found within 2 s after a change. Both were worse for the HPD condition. These types of problems could potentially slow work performance and lead to mistakes related to misunderstandings in communication. In addition to this, the accuracy to which individuals could track target objects was also diminished in the HPD relative to the no HPD condition. This could be especially the case around critical listening periods where error increases, and slowed adjustment to changing commands is apparent (even when commands are comprehended; see Figure 5a).

When a monetary reward was on the line, listening in the no HPD condition was preferred. This was a strong preference with almost 85% of the sample choosing this option. While it needs to be acknowledged that sounds were presented at safe levels, and that conditions were not referenced to subjects by their HPD association, this data does objectively show that performance relates to choice. We certainly expect that when a trade-off of safety is introduced, choice would be less biased towards unprotected listening. Even so, the data suggest a bias that needs to be overcome within a real-world work environment.

### Work relevance

That HPD wearing worsens performance is incredibly important to workers. We know from surveys and focus groups of actual worker samples that performance problems are a commonly reported reason for non-use (Boissinot et al., 2022; Casali et al., 2009; Laitinen, 2005; Rabinowitz et al., 2018). In one assessment of hearing conservation program effectiveness over multiple metal manufacturing sites, it was found that sites in which workers more frequently reported interference with job performance also had higher rates of hearing loss (Rabinowitz et al., 2018). This was the case even when site noise level was taken into account. In musicians, where ongoing adaptation to auditory feedback is critical, 65% of one sample reported not wearing HPDs as a performance advantage (Olson et al., 2016; also, see Couth et al., 2022). It is clear that HPD-related performance detriments matter to workers. The choice to not wear an HPD during noise exposure will lead to hearing loss in these individuals.

It is often advocated that workers need more training with materials that highlight awareness of occupational HL as a problem, that the problem is relevant, that the problem should be important to the worker at the individual level, and that HPD wearing can prevent the problem (e.g., see Stephenson, 2009). In actuality, there is very little evidence that such knowledge interventions work. Much of the evidence cited in support for such approaches comes from what workers say they will do, or what they presently believe, after being told what they should do and what they should believe. For instance, Stephenson (2009) reported that 31% of a worker sample agreed or strongly agreed with the statement, “I can understand speech well enough to do my job while I am wearing hearing protectors” before training. After training, this agreement improved to 71%. This may initially seem promising, but data and methods like this do not provide objective measures of choice. They introduce a strong experimenter demand characteristic that is likely to bias answers that workers give. Workers can easily infer the desirable response from the experimenter and choose accordingly (Zizzo, 2010). The data from such studies also runs counter to decades long studies that demonstrate very little change in HPD use rates or occupational HL when such training is implemented (Brennan-Jones et al., 2020; Green et al., 2021; Tikka et al., 2017). We believe that training should include aspects that address performance issues raised by wearing an HPD. These aspects could be included along with training meant to convince workers that HPD wearing is important.

What could such training entail? At least two ideas have been put forth. The first is to take advantage of the number of different HPD options (Casali et al., 2009; Gallagher et al., 2014; Stephenson, 2009). There are HPDs with a variety of different attenuation profiles, active noise reduction capability, and sound restoration technology. Part of training could involve finding an individual’s best performing HPD. For instance, workers could try out a number of devices at work until one is found that suits their performance needs. This could also aid with comfort issues that impact willingness to wear an HPD. Another option is to address issues in auditory perception with an auditory training regimen. Experience modifies auditory perception abilities; a phenomenon referred to as *perceptual learning* (for review, see Irvine, 2018; Wisniewski et al., 2017; Wright and Zhang, 2009). Training regimens aimed at facilitating perceptual learning have become increasingly popular for addressing degraded auditory perception in clinical abnormalities (e.g., for individuals adjusting to cochlear implants; Khayr et al., 2024). One recent study from our lab showed that training with a simulated HPD could improve speech comprehension and lead to less listening effort while wearing an HPD (Wisniewski and Zakrzewski, 2020; also see Casali and Robinette, 2015; McClain and Wisniewski, 2026). It could be very easy to implement listening training as part of a hearing loss prevention plan at work. Training could also be made to be entertaining for workers by presenting interesting audio material (e.g., podcasts; cf. Zhang and Samuel, 2014) filtered through a simulated HPD. This type of listening training, combined with tailored HPD selection, could improve worker listening experiences, performance, and make it more likely that workers protect themselves with an HPD. Of course, all of this remains speculative until studies are done that implement perceptual training and HPD selection processes in samples of real workers.

### Caveats and considerations

We did not observe any significant correlation between individual performance and the value individuals placed in the choice to listen in the HPD or no HPD condition. It may be that no relationship exists; however, we believe this to be unlikely given that a vast majority of our subjects made an actual choice to listen without a simulated HPD (i.e., the condition in which they performed best). The lack of an effect could be due to insensitivity of the choice measure. Large individual differences in how participants use the scale of options, or systematic biases (e.g., some with bias to select most reward no matter what), could mask an underlying performance relationship. Another possibility is that such a relationship is masked by strong effects induced by the type of HPD we simulated. We chose a common passive HPD (Howard Leight Max) with a strong attenuation profile. It is likely that an HPD with a less drastic attenuation profile would weaken the performance detriments that are produced by wearing; and as a result lead to HPD choices that are less biased to unprotected listening. It also needs to be considered that we have yet to test simulation of different HPDs with this new paradigm. It is possible that different passive HPDs or active HPDs yield less worsening on auditory situational awareness and concurrent sensorimotor tracking. It will be important to measure how use of different HPD types impacts the likelihood of making the choice to wear an HPD.

Some readers might take issue with the fact that our measure of choice to wear an HPD does not perfectly mimic such a choice in a real occupational scenario. High noise is not used (there are no real safety consequences), a real HPD is not used, there is no risk to losing a job, and there are no employer rules to wear an HPD. Though these real-life features are likely important, many are difficult to ethically work into a lab-based study. Before exploring these higher risk scenarios in actual occupational settings, it is necessary to conduct lab studies like this that demonstrate effects with objective, rather than subjective survey measures. There is reason to expect that some features of a real-life scenario (e.g., risk of being fired for poor performance) would make the performance effects on choice even stronger.

Several characteristics of our sample may limit the generalizability of the results. First, the sample was primarily made up of females. There is some evidence that attitudes about HPD use may differ between men and women (McCullagh et al., 2016). A better balance of sex in future studies could allow us to explore potential sex differences that cannot be well addressed with our current sample. Our sample was primarily younger age college students that self-reported normal hearing (not confirmed with audiological testing). Participants could have been biased to report normal hearing, or insensitive to mild/hidden hearing loss and middle ear conditions. Also a potential issue is that the generalizability of our results to older worker samples could be limited. Nevertheless, there is still HPD relevance for this age demographic. In a 2015 study of college-aged students’ and choice to wear hearing protection, it was found that only ∼41% reported using hearing protection. This was the case even though ∼80% of the sample reported being exposed to high noise (Balanay and Kearney, 2015). Hearing loss symptoms have been increasing in younger populations (Niskar et al., 2001), so it may become increasingly important to study the younger age demographic. It may also prove worthwhile to include audiological testing in future studies with younger samples in order to examine how individual differences in hearing levels relate to choice and performance.

It needs to be acknowledged that we used a simulated HPD condition rather than actual HPDs. There are clear advantages to this. First, with actual HPDs, differences in subject fit, comfort, or willingness to insert an HPD could influence choice. We did not want these issues to be confounding factors in relating performance to choice. Second, change in fit over time in the experiment (or differences in fit) has the potential to corrupt HPD versus non-HPD comparisons and any evaluations of the relationship between performance and choice (e.g., choice could be related to fit rather than performance under difficult listening). With the HPD simulation, we can be sure that all listeners receive the same acoustic conditions. Third, the simulation allowed us to avoid potential biases based in listeners’ beliefs about hearing protection (e.g., “protecting my hearing is good, so I should choose to wear it”). Though beliefs and biases are interesting, this study was focused on performance. By using the HPD simulation, we could refer to the HPD condition as “quiet.” Thus, we shortcut biases. Nevertheless, generalizability to actual HPD wearing has not been established here. Future research that measures performance and choice along with other variables related to comfort, fit, and preexisting bias with actual HPDs will be useful in establishing this generalizability. Similarly, real worker populations should also be used. Now that a proof-of-concept lab-based study with simulated hearing protection shows a relationship between performance and choice, it is more justifiable to invest resources into such studies.

## Conclusion

The current study shows that: (1) HPD wearing hurts auditory situational awareness relevant for work-like tasks, (2) performance worsening produced by HPD wearing extends to nonauditory aspects of multitasking (e.g., concurrent sensorimotor performance), and (3) that preference for listening in an unprotected condition is strong when performance matters most. Occupational HL rates and HPD use rates are not changing for the better at an acceptable pace. Addressing the performance issues that arise while wearing an HPD could be one key to solving the problem.

## Data Availability

The raw data supporting the conclusions of this article will be made available by the authors, without undue reservation.

## Footnotes

1 None of the participants in this study were given hearing tests or audiological examinations. We decided not to give these tests because it was expected that HL would be minimal in such a young sample. Also, adding these procedures would significantly impact experiment time, having an impact on fatigue and on the ability to collect a sufficient sample size.
